# Developing a diagnostic checklist of traditional Chinese medicine symptoms and signs for psoriasis: a Delphi study

**DOI:** 10.1186/1749-8546-8-10

**Published:** 2013-05-12

**Authors:** Xuesong Yang, Virasakdi Chongsuvivatwong, Edward McNeil, Jianzhou Ye, Xiaoyong Ouyang, Enpin Yang, Hutcha Sriplung

**Affiliations:** 1 Dermatology Department, Yunnan Provincial Hospital of Traditional Chinese Medicine, Kunming, 650011, China; 2 Epidemiology Unit, Faculty of Medicine, Prince of Songkla University, HatYai, Songkhla, 90110, Thailand

## Abstract

**Background:**

Psoriasis is a chronic inflammatory skin disease with a genetic basis. Its ill-defined causes make it difficult to diagnose. This study aims to develop a diagnostic checklist for psoriasis classification in the context of traditional Chinese medicine.

**Methods:**

A Delphi study was conducted with three rounds by a panel of 16 dermatology experts to develop a checklist for traditional Chinese medicine symptoms and signs of psoriasis. Dermatology experts in psoriasis research, nine in Yunnan and seven in Beijing, were selected as the expert panel. The initial list of symptoms and signs in psoriasis was developed by reviewing the literature retrieved from Chinese and English journals. Experts rated each item of the list on a 5-point Likert scale. The list was revised and re-evaluated in the same manner for a total of 3 rounds before it was finalized.

**Results:**

One hundred and thirty items were extracted from the literature review. After three rounds of expert ratings, 96 items were retained with eight domains: color, type and shape of skin lesion, physical expression, tongue and coating, pulse, associated factors, and living environment. Intraclass correlation coefficient and Kappa statistics indicated an inter-rater agreement in the final checklist.

**Conclusion:**

A checklist containing 96 items in 8 domains was developed for psoriasis diagnosis using traditional Chinese medicine symptoms and signs.

## Background

Psoriasis is a chronic, inflammatory, hyper-proliferative and immunologically mediated skin disease with a genetic basis [1]. It is characterized by erythema, defined as scaly plaques with itching and bleeding [2]. Psoriasis prevalence ranges from 0.6% to 4.8% in all age groups worldwide [3,4] and psoriatic arthritis (PsA) affects 6–39% of all psoriasis patients [5-7]. The variation in psoriasis prevalence with geographical and ethnic factors suggests an influence of the physical environment (*e.g*., climate) patterns or other exposures in the development of psoriasis [8-10].

In Western medicine, psoriasis treatments can be any one or a combination of the three main modalities: topical treatments with steroids or other agents, UV therapy with or without light-sensitizing medication and systemic medications, such as retinoids, and cytotoxic drugs [11,12]. Some psoriatic patients (51%) may also opt to use alternative therapies, such as herbal remedies, vitamin supplements, and dietary manipulation [13], and traditional Chinese medicine (TCM) [14].

According to TCM diagnosis [15], psoriasis (白疕, *bai bi* or white mange) [16] is considered to be due to (1) the invasion of pathogenic wind that incubates in the yin and blood; or (2) the accumulation and stagnation of *qi* (vital energy) and blood caused by emotional upset. It may also be caused by impairment or disharmony in the functioning of other organs and energy pathway channels [17]. In general, topical treatment, oral medication, and advice on lifestyle and environment are prescribed to relieve the lesions and the impairment or disharmony of the body.

TCM treatment is based on Zheng composition rather than disease definition [18,19]. For the same disease, there are varieties of syndromes related to different climates and geographical factors [20].

Developing a consensual checklist of TCM symptoms and signs to aid identifying and classifying psoriasis would be useful for TCM clinical practice and scientific research. The Delphi method is a consensus methodology that attempts to assess the extent of agreement (consensus measurement) and to resolve disagreement (consensus development) in medical and health service research [21,22]. The Delphi technique can be performed in 3 stages. First, the opinions of a group of experts are sought based on an anonymous self-administered questionnaire to eliminate the influence of peer pressure. Second, a feedback system is used to allow the respondents to compare their opinion against a statistical summary of the whole group. Finally, this process is repeated until the opinions are stable [23,24]. Thus, using the Delphi method, experts can participate without geographic limitations, offer their opinions independently and confidentially without face-to-face meetings and change their opinion based on the systematic feedback from the results of the previous rounds in consecutive stages of the process [25].

This study aims to develop a consensual checklist using the Delphi method for psoriasis classification by TCM symptoms and signs.

## Methods

### Selection criteria

The study was conducted in the dermatology departments of TCM hospitals in Beijing and the Yunnan province of China. The departments in Dongzhimen Hospital and Yunnan Provincial Hospital of TCM, have collaborated for more than 10 years. Beijing was selected as a representative for northern China where much research on psoriasis has been carried out. Yunnan Provincial Hospital of TCM is the only psoriasis research center in Yunnan and was selected to represent southern China. TCM experts were recruited from five major TCM hospitals in Beijing and one in Yunnan. The hospitals in Beijing were Guang’anmen Hospital, China Academy of Chinese Medical Sciences, China-Japan Friendship Hospital, Dongzhimen Hospital Affiliated to Beijing University of Traditional Chinese Medicine, Dongfang Hospital Affiliated to Beijing University of Traditional Chinese Medicine, and Beijing Hospital of Traditional Chinese Medicine. Yunnan Provincial Hospital of TCM was the selected one in Yunnan.

All participating experts were engaged in psoriasis research for at least ten years, and had each published at least ten research articles on psoriasis. Their academic position was associate professor or higher. Twenty dermatology experts were recommended by the steering committee on a videoconference based on their experience in psoriasis research and four were later excluded, as they did not have sufficient research publications. Finally, seven and nine experts were recruited from Beijing and Yunnan, respectively.

The selection committee was formed by key members of the TCM Dermatology Association (two from Beijing and two from Yunnan) in psoriasis research, which formulated the panel and item selection criteria. These four professors were the most respected leaders in research and administrative authorities in China. They had a mean working experience in psoriasis of 20 years with at least 15 publications. Two of them were the current and ex-chairmen and two were the present vice chairmen in the dermatology branch of the China Association of Chinese Medicine.

### Literature review

A literature review was performed to define the initial list of symptoms and signs of psoriasis. The keywords were ‘psoriasis in TCM’, ‘screening and diagnosis of psoriasis in TCM’, ‘different research/schools of psoriasis in TCM in China’. PubMed (http://www.ncbi.nlm.nih.gov/PubMed/) was the source of international publication retrieval. China National Knowledge Infrastructure (CNKI, http://www.cnki.net/), Wanfang data and VIP knowledge communities databases (http://www.wanfangdata.com.cn/) were the sources of articles published in Chinese. Articles were included if they were published between 1970 and 2010. Case reports and articles based on subjective experience and treatment were not excluded since the aim of the review was to identify symptoms, signs, and Zhengs associated with psoriasis.

### Checklist design

The initial checklist was derived from the literature review. The experts rated each item in the checklist with one of the following scores: (1) strongly disagree; (2) disagree; (3) neutral; (4) agree; and (5) strongly agree. The items covered 8 domains, including color, characteristic, and shape of the skin lesions, associated factors, physical expression, tongue and its coating, pulse, and living environment.

### Seeking expert consensus

The survey was conducted in three rounds from July to September 2010. Individual face-to-face interviews were held in the first round. The objective of the study, content of the questionnaire, and scoring method were explained to the experts. The experts scored each item in the checklist and wrote down their comments. They could also add more items if they thought it appropriate. The mean ± standard deviation (SD) of each item from the first round was computed. Items with a mean score below three were removed from the list used for the following round.

In the second round, the selected items and their first round mean ± SD values as well as the newly added items were compiled and sent confidentially to each expert by email. The expert was asked to re-rate the items with a full awareness of their previous rating and mean score of the items rated by all experts. New items added by any of the experts from round 1 were rated in round 2.

The new mean scores of items were computed, and items with a mean score less than three were removed. The remaining items together with their means ± SD from round 2 were sent by email to all experts. Items with a mean score of more than three in the third round were retained to form the final checklist.

### Statistical analysis

Descriptive statistics of all items were summarized. Intraclass correlation coefficient (ICC) was used to assess the agreement and consistency among the experts across the three rounds. Fleiss-Cohen (quadratic) weighted Kappa statistics, in which the weight score was calculated based on a quadratic (square) loss function, were also used to explore inter- and intra-rater agreement [26]. Agreement was considered “very good” if Kappa reached 0.81 or higher, “good” if between 0.61 and 0.80, “moderate” if between 0.41 and 0.60, “fair” if between 0.21 and 0.40 and “poor” if equal to or less than 0.20. The effects of variation of both rounds and experts on the scores were examined with analysis of variance (ANOVA) using R software version 2.12.1 (Vienna, Austria). Hierarchical cluster analysis was performed to examine closeness among various experts’ ratings, and was expressed as a dendrogram. The province name was used to label each branch of the dendrogram to see whether the opinion was clustered by province. All statistical analyses were carried out using R software version 2.12.1, a free open source software environment for statistical computing and graphics available from R-project.org website [27], the Epicalc package was downloaded from the CRAN website (cran.r-project.org) [28] for Kappa statistics, and the psy package was also freely available at the CRAN website for ICC [29].

## Results

Sixteen experts were recruited, seven from Beijing and nine from Yunnan provinces. The rating expectative panel of dermatologists in Beijing consisted of four males and three females, while in Yunnan it comprised five males and four females. Two experts were professors and five were associate professors in Beijing while seven experts were associate professors and two were professors in Yunnan province. The total age (mean ± SD) of the experts was 47.7 ± 4.9 years; 46.6 ± 4.8 years for those in Beijing, and 48.6 ± 5.2 years for those in Yunnan province.

Among 102 reports located in the literature, none was excluded since all mentioned symptoms, signs and Zhengs related to psoriasis. One hundred and thirty items were extracted and grouped into one of the following 8 domains: (1) skin lesion color; (2) type of skin lesion; (3) shape of skin lesion; (4) associated factors; (5) physical expression; (6) tongue and its coating; (7) pulse; and (8) living environment. All domains except the last were present in the initial checklist of the first round of the Delphi study, the 8th domain was added on expert suggestion in the second round.

### Checklist

Table 1 summarizes the number of items of symptoms and signs in each round. One hundred and thirty items were rated in the first round by the experts. Eighty six items with a mean score greater than three remained after the first round and 24 new items were added by the experts. A total of 110 items were rated in the second round. A total of 105 items remained at the start of the third round. Ninety six items with mean score greater than or equal to three were retained in the final list (Table 2).

**Table 1:** Number of items at the end of each round

**Domain**	**First round**			Second round*		**Third round** *		**Final**
	**Initial**	**Removed**	**Added**	**Remaining**	**Removed**	**Remaining**	**Removed**	—
1. Color of skin lesion	5	—	—	5	—	5	—	5
2. Type of skin lesion	11	5	—	6	—	6	—	6
3. Shape of skin lesion	21	5	—	16	—	16	—	16
4. Associated factors	9	1	4	12	—	12	2	10
5. Physical expression	56	28	16	44	4	40	7	33
-Diet indulgence and stool	10	5	—	5	—	5	—	5
-Mental irritation	9	7	—	2	—	2	—	2
-Itching	6	—	—	6	—	6	—	6
-Fever and chills	7	5	—	2	—	2	—	2
-Urine feature	5	4	—	1	—	1	—	1
-Joint and muscle pain	6	4	—	2	—	2	—	2
-Mouth	5	—	—	5	—	5	—	5
-Sleep	4	1	—	3	1	2	—	2
-Complexion	4	2	—	2	—	2	1	1
-Throat	—	—	5	5	2	3	—	3
-Menstruation	—	—	9	9	1	8	5	3
-Nail	—	—	2	2	—	2	1	1
6. Tongue and its coating	16	1	—	15	—	15	—	15
7. Pulse	12	4	—	8	1	7	—	7
8. Living environment	—	—	4	4	—	4	—	4
Total	130	44	24	110	5	105		96

* No additional item.

**Table 2:** List of items in the final round

**Domain**	**Symptoms and signs**
Skin lesion color	Full red, red, garnet, pink, darker skinned
Type of skin lesion	Papule, scaling, crust, pustule, cracking, erythema
Shape of skin lesion	Scattered, guttate, ostaceous, plaque, map shape, generalized lesion, annular, thickness, hyperpigmentation, depigmentation, rough surface, infiltration, thin scale, thick scale, easy scaly exfoliation, dry scale
Associated factors	Trigger factors: overexertion, depression, stirring
	Aggravating factors: smoking, drinking alcohol, hot water, stimulating medicine, infection, during menstruation, postpartum
	predilection diet: heavy and greasy, pungent, cold and raw
	Stool: less stool, constipation
	Mental irritation: sense of distress in the chest, anxiety and irritability
	Itching degree: severe, mild, slight, absent
	Itching frequency: continuous, intermittent
	Fever: fever, hot palms, soles and heart
	Urine: scanty dark urine
	Muscle and joint: muscles and joint pain, stiffness with bending limitation
	Mouth: dry mouth and thirsty, dry mouth and not thirsty, bitter taste, fetid breath, sticky taste
	Sleep: insomnia, frequent dreams
	Complexion: flushed
	Nail manifestation: nails not lustrous
	Menstruation: scanty menstruation, crimson color, blood clot
	Throat: sore throat, redness of pharyngeal portion, tonsil suppuration
Tongue and its coating	Substance: pale, red, maroon, dark purple tongue or tongue with petechiae, thin delicate, enlarged tongue fissured
	Coating: white, yellow, greasy, glossy, rough, less, peeling, sublingual varicose veins and bluish purple
Pulse	Moderate, slippery, rapid, string, deep, thread, hesitant
Living environment	Humid, dry, hot, cold

### Agreement of experts in the three rounds

Table 3 summarizes the analysis of intra- and inter-rater agreements. The mean weighted kappa statistics for the scores of all items rated by the same expert in the first and the two executive rounds showed moderate agreement (0.34 and 0.42, respectively) whereas agreement between the second and third rounds was almost perfect (0.96). Inter-rater agreements (last column) were poor (0.12 to 0.18) in all three rounds. Intra-class correlation coefficients were between 0.23 and 0.29, indicating that the level of agreement among experts was low.

**Table 3:** Agreement between rounds within the same raters and between different pairs of raters in the three rounds

**Agreement status**	**Same rater, different round (mean ± SD)**			**Pairs of raters (mean ± SD)**
	**First round**	**Second round**	**Third round**	
Kappa^1^ statistics				
First round	1.0	—	—	0.12 ± 0.12
Second round	0.34 ± 0.20	1.0	—	0.16 ± 0.13
Third round	0.42 ± 0.19	0.96 ± 0.06	1.0	0.18 ± 0.12
Intraclass correlation				
coefficient (ICC)				
Agreement	0.23	0.26	0.26	—
Consistency	0.27	0.29	0.29	—

^1^ Quadratic weighted kappa statistics.

SD, standard deviation; ICC, intraclass correlation coefficient.

Table 4 summarizes the analysis of variance of scores rated by experts in different rounds. ANOVA shows the magnitude of the variance of the scores determined by expert and round. We observed a high F-value that produced a low *P* value of 0.000003 (highly significant) for the effect of experts, whereas the effect of round within the same individual expert on the variance of scores was low and non-significant (*P* = 0.95). This suggested that each expert had different opinions from their peers but had a consistent opinion within themselves.

**Table 4:** ANOVA analysis of scores among experts and rounds of the Delphi process

	**Df**	**Sum square**	**Mean square**	**F-value**	**P (> F)**
Expert	15	27282.3	1818.8	7.020	0.000003
Round	2	24.7	12.3	0.048	0.95
Residuals	30	7772.7	259.1		

Df: degree of freedom.

F-value is the division of mean square of expert or round by the residual mean square.

P(>F) is the *P* value that the effect of expert panel or round on the mean scores can be rejected against the null hypothesis.

**Figure 1:**
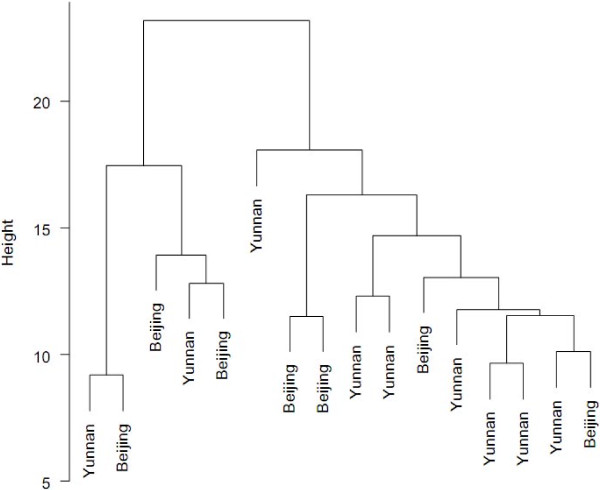
Dendrogram showing clusters among raters.

Figure 1 shows a dendrogram from hierarchical cluster analysis of experts based on their score in the final set of items. The ratings of the experts from Yunnan and Beijing were not significantly different.

## Discussion

From the literature and Delphi method, 96 items of TCM symptoms and signs were selected for psoriasis classification. Although the level of agreement among the experts was not perfect, a final checklist was developed.

Type, color and shape of the skin lesion were rated with the highest scores among the 8 domains. This was consistent with the Psoriasis Area Severity Index (PASI), which places strong weight on skin-related items [30].

Smoking and drinking are generally referred to as external pathogenic factors. Both past and current smokers have a higher risk of developing psoriasis and the risk increases with duration, intensity and pack-years of smoking [31]. These factors were included by the experts in our study.

Physical expression items contributed to as many as 30% of all items. Two viscera contributed seven items: *Spleen*-related items linked to gastrointestinal symptoms [32], and Liver-related items linked to mental health [33]. The *Spleen* group was not as strong as the *Liver* group in psoriasis symptoms based on TCM.

Itching is an important symptom in psoriasis that correlates with the severity of disease and is experienced by almost 85% of patients [34,35]. It causes anxiety, distress and worsens the patients’ mental health [36]. In the final round, the number of itching items was the highest among physical expression items.

Only two orthopedic items, joint and muscle pain and stiffness with bending limitations, were included by the experts. This may be because arthritic complications of psoriasis are often treated by rheumatologists rather than dermatologists [37]. Symptoms of PsA may closely resemble other diseases including rheumatoid arthritis.

Tongue and its coating are often examined in TCM. Up to 8.2% of fissured tongue and 5.6% of geographical tongue were observed in psoriatic patients in Iran [38]. Tongue coating is associated with the depth of skin involvement. Tongue substance reflects excess or deficiency of *qi*, *blood* and *viscera*[39]. The manifestations vary over the long-term course of psoriasis. Seven tongue substances and 8 tongue coatings were included in the final checklist.

Pulse palpation is an important method to classify the syndrome of psoriasis. Slippery or rapid pulse reflects blood heat. Hesitant and deep pulse reflects blood stasis [40]. Seven types of pulse and its manifest frequency were included for psoriatic patients.

Certain types of symptoms and signs retained by the experts in the final checklist coincided with various known psoriasis risk factors, *e*.*g*., *streptococcal* infection, sore throat and female hormones [41,42].

Environmental factors can influence psoriasis, *e*.*g*., coldness and low humidity. Psoriatic patients can recover more quickly in a hot or humid environment [43]. The presence of these four major environmental factors are related to external etiology factors, and are manifest in the symptoms and signs of psoriatic patients.

There was no obvious evidence of clustering of opinions by geographic setting of the experts. However, a strong conclusion of the similarity of their opinions could not be made since there were a limited number of expert informants. This standardized checklist was approved by psoriasis experts in two different regions of China and might be used throughout the whole of China.

The mean weighted Kappa statistics for the same rater in different rounds showed that raters agreed with themselves in the consecutive rounds. For example, a Kappa value of 0.34 between the first and the second rounds and 0.42 between the second and third rounds (Table 3). When the third round was reached, agreement with the second round was very high and stopping the Delphi process was justified since it was unlikely there would be further changes in the experts’ opinion even though it may have differed from that of the others. The low Kappa statistics in the last column of Table 3 and the low ICC values confirmed that experts did not agree with each other for many items. This implies that experts were unlikely to have achieved a high consensus in opinion even if more rounds had been performed [44]. Recommendations have previously been made for the number of rounds of the Delphi technique [45]. Four Delphi rounds were suggested by Sumsion [46]. However, two to three rounds are currently used, to avoid fatigue of the respondents [47]. Our findings confirm that more than three Delphi rounds would probably not be of further benefit. Table 4 shows the non-significant effect of round number on experts’ opinions, and demonstrates that participants did change their ideas a little when they saw the results in the first round but did not change their ideas in the subsequent rounds and overall agreement could not be better as rounds increased.

Even though Delphi is a good study design for achieving consensus among experts, a true consensus might not be met since it is a manipulated consensus based on a compromised position [48]. Even though they had the freedom to make comments, experts were unavoidably forced to follow the structural domains we proposed in the first round. It was also suggested that experts should be selected from a wide spectrum of vested interest [49]. However, we selected only those from Beijing and Yunnan on the basis of convenience and budget constraint. However, the dendrogram (Figure 1) shows that experts’ ideas were independent of their geographical working location. Even though some Delphi studies have recruited very large numbers of participants for the panel, some authorities suggest a panel of 7 to 12 and some suggest up to 10 to 50 at most [45,50]. As mentioned above, fixation of experts’ idea was reached at the third round, and having too many informants in a panel may not be a good strategy to improve agreement among them.

The development of a checklist from a consensus of well-recognized experts in psoriasis in China, allows further research studies and clinical practice to be standardized. Furthermore, based on a standard list of symptoms and signs, diagnosis and treatment of psoriatic patients may be unified and variations in clinical practice among TCM doctors can be minimized.

## Conclusion

A checklist containing 96 items of 8 domains was developed for classifying psoriasis with traditional Chinese medicine symptoms and signs.

## Competing interests

The authors declare that they have no competing interests.

## Authors’ contributions

XSY, VC, HS, JZY, EPY and XYOY designed and conducted the study. XSY, VC and HS wrote the manuscript. EM and XSY performed the statistical analysis. All authors read and approved the final manuscript.
